# Microbiological transglutaminase: Biotechnological application in the food industry

**DOI:** 10.1515/biol-2022-0737

**Published:** 2023-09-30

**Authors:** Vitaliy Kolotylo, Kamil Piwowarek, Marek Kieliszek

**Affiliations:** Department of Food Biotechnology and Microbiology, Institute of Food Sciences, Warsaw University of Life Sciences – SGGW, Nowoursynowska 159 C, 02-776 Warsaw, Poland

**Keywords:** transglutaminase, mTG, food industry, enzyme, microorganisms

## Abstract

Microbial transglutaminases (mTGs) belong to the family of global TGs, isolated and characterised by various bacterial strains, with the first being *Streptomyces mobaraensis*. This literature review also discusses TGs of animal and plant origin. TGs catalyse the formation of an isopeptide bond, cross-linking the amino and acyl groups. Due to its broad enzymatic activity, TG is extensively utilised in the food industry. The annual net growth in the utilisation of enzymes in the food processing industry is estimated to be 21.9%. As of 2020, the global food enzymes market was valued at around $2.3 billion USD (mTG market was estimated to be around $200 million USD). Much of this growth is attributed to the applications of mTG, benefiting both producers and consumers. In the food industry, TG enhances gelation and modifies emulsification, foaming, viscosity, and water-holding capacity. Research on TG, mainly mTG, provides increasing insights into the wide range of applications of this enzyme in various industrial sectors and promotes enzymatic processing. This work presents the characteristics of TGs, their properties, and the rationale for their utilisation. The review aims to provide theoretical foundations that will assist researchers worldwide in building a methodological framework and furthering the advancement of biotechnology research.

## 1 Introduction

Protein is not only one of the main macronutrients but also a key structural component of many food products. In recent decades, due to concerns about global food shortages (including population growth), there has been a growing interest in the advanced utilisation of food proteins [1]. The application of enzymes in food processing, from the producers’ perspective, should be associated with commercial benefits, and from the consumers’ perspective with improved product quality. The availability of enzymes on a large scale and at reasonable prices has led the food industry to reconsider their use in food processing. Currently, advancements in biotechnology and genetic manipulation have created new prospects for efficient enzyme production, leading to the emergence of innovative technologies for their utilisation [2].

To meet the diverse food needs of consumers, enzymatic protein modification techniques are recommended, which have several advantages over chemical modification, including high reaction specificity, the low occurrence of side reactions, and the absence of the need for high temperature, pressure, or chemical solvents. Hydrolytic enzymes with a cleaving action, such as proteases and amylases, are commonly used. The subject of this review is transglutaminase (TG; protein-glutamine γ-glutamyltransferase), which catalyses acyl transfer reactions, introducing covalent cross-links between proteins and forming high-molecular-weight polymers [3].

The market for industrial enzymes has exhibited steady growth over the last six decades, expanding from around USD 0.31 billion in 1960 to a substantial USD 6 billion in 2020. Within the numerous industries where enzymes have found utility, the food sector commands the largest share, with approximately 55% of industrial enzymes finding applications in various segments of the food industry, including baking, beverages, brewing, dairy, oil refining, food packaging, and fruit and vegetable juice processing. As of 2020, the global food enzymes market was valued at around USD 2.3 billion, constituting roughly 40% of the total value of the industrial enzymes market. Predictions indicate that by 2026, the global food enzymes market will exceed USD 3.3 billion. This projection suggests that microbial enzymes with uses in the food industry will continue to experience demand. As of September 2021, the microbial transglutaminase (mTG) market was estimated to be around $200 million USD on a global scale. It is worth noting that these figures are subject to change over time due to shifts in market dynamics, technological progress, and other influencing factors [4].

Until the late 1970s, proteases were the main class of enzymes used for protein modification. However, with the discovery of TG, the diversity of this technology expanded [5]. The topic of TG holds a specialised yet important position in today’s times for several reasons. TG is an enzyme with a specific role in biochemical processes, playing a crucial role in various physiological functions. Studying and understanding the function of this enzyme is of paramount importance for a better grasp of different aspects of biology and biochemistry. TG catalyses the formation of inter- and intramolecular covalent bonds between proteins [6]. The covalent cross-links generated by this enzyme can be utilised to modify the physical and chemical properties of proteins, such as solubility, water-holding capacity, thermal stability, emulsifying and foaming properties, viscosity, elasticity, and gelation [7]. Many proteins can be modified, and some of the most commonly modified include milk casein, whey proteins, soy globulins, wheat gluten, meat myosins, and egg proteins [6,8]. TG finds broad application in the food industry. It acts as an ingredient that impacts the texture, durability, and quality of food. Research on different types of TG, including microbial variants, provides new insights into its potential use in food production. TG can be utilised to create products with improved sensory and technological properties, and it holds the potential to serve as a tool for reducing food waste. In addition to its widespread use in the food industry, the increasing interest in TG has led to the discovery of its advantages in other fields such as medicine, biomedical engineering, materials science, textiles, and leather processing [9], where it can be used for protein modification, biomaterial production, or gene therapy.

Finally, the advancement of new research methods and technologies enables more precise studies on TG and the identification of new variants. This contributes to the development of knowledge about the enzyme and its potential, which can lead to the discovery of new applications and innovative solutions in various fields of science and industry.

In summary, the topic of TG holds a specialised yet important position in the present era due to its biological significance, broad application in the food industry, potential in other fields, and the development of new research technologies that allow the exploration of its unique properties and uses.

## 2 Characterisation of the protein-glutamine γ-glutamyltransferase enzyme

TG is a widely occurring enzyme belonging to the transferase group. The activity of TG was first observed by Clarke et al. [10] when they extracted the enzyme catalysing the transamidation reaction in the liver of *Cavia porcellus*. 2 years later, in 1959, a team of researchers led by Waelsch assigned the name “transglutaminase” to the aforementioned enzyme in order to differentiate this specific enzymatic activity from other enzymes with similar properties [11]. According to the International Union of Biochemistry and Molecular Biology Nomenclature Committee, TG is assigned the EC number 2.3.2.13, which belongs to aminoacyltransferases (EC 2.3.2), and these enzymes belong to acyltransferases (EC 2.3) (BRENDA – The Comprehensive Enzyme Information System). In the available scientific literature, one can come across the systematic name (protein-glutamine γ-glutamyltransferase) as well as synonyms for this enzyme, such as fibrinoligase. However, the most commonly used name is TG.

TGs are a large family of related and ubiquitous enzymes that catalyse post-translational modifications of proteins in plants, animals, and microorganisms [8,9]. TG is a transferase enzyme responsible for the transfer of an acyl group (or acyl moiety) between the γ-carboxamide group of glutamine residues bound to peptides (acyl donors) and various primary amines (acyl acceptors), including the ε-amino group of lysine residues in proteins [9,12]. When a free lysine residue acts with glutamine as an acceptor for the acyl group of the protein substrate, the initial protein is enriched with this exogenous amino acid for humans. TG can initiate inter- and intramolecular cross-linking reactions between protein molecules by transferring the acyl group to a lysine residue that is associated with a polypeptide or protein [13]. In the absence of amino acid substrates, water acts as an acyl acceptor, and the γ-carboxamide groups are deamidated to glutamic acid residues, a reaction known as deamination. Additionally, the enzyme catalyses the addition of free amines to proteins through conjugation with glutamine residues [12,14].

**Figure 1:**
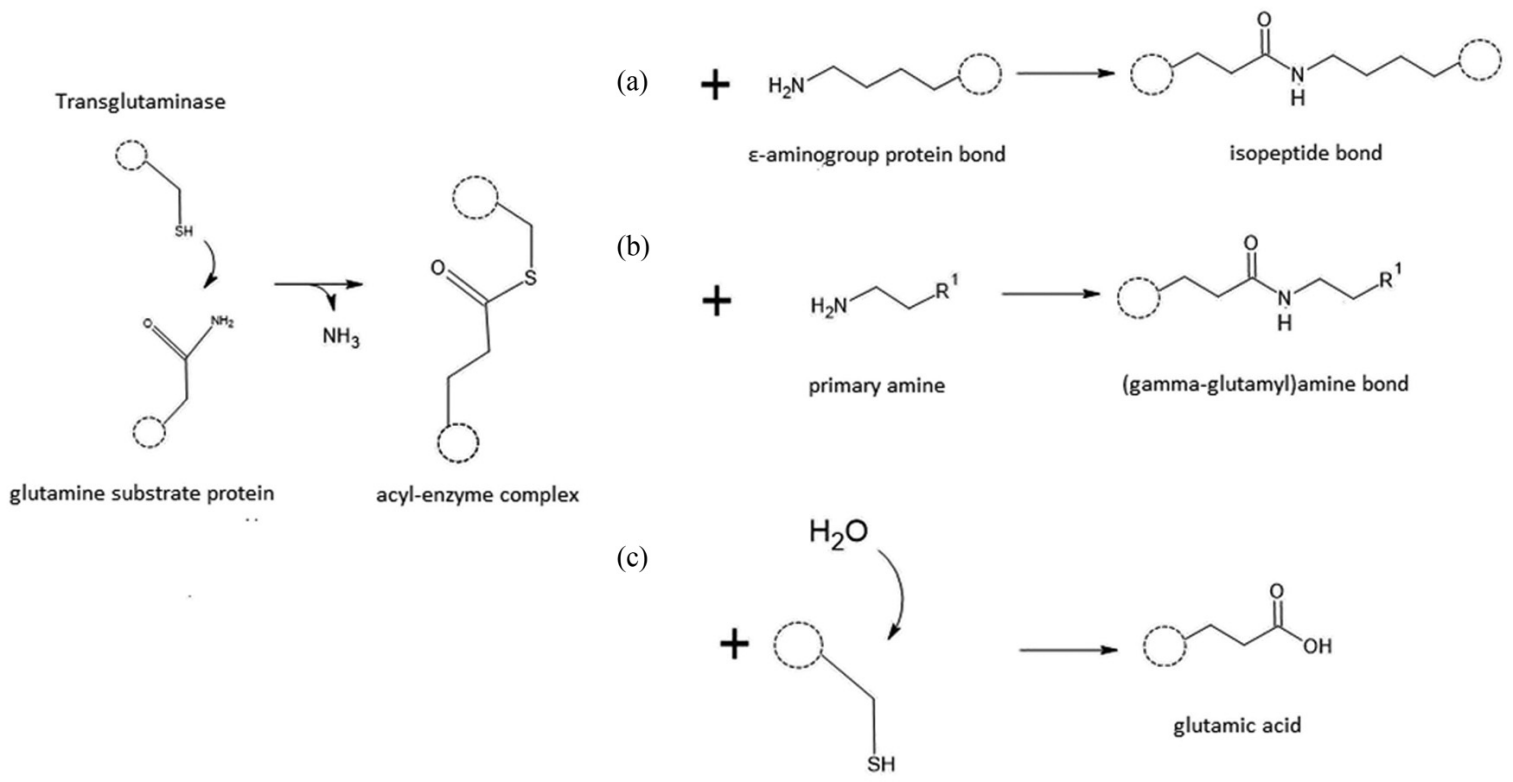
Reactions catalysed by TG.

According to Alvarez et al. [15], a characteristic feature of TGs is the active site, which consists of a triad of amino acids: cysteine, asparagine, and histidine. This catalytic triad in the enzyme’s active site interacts with the bound glutamine in the polypeptide chain, resulting in the formation of an acyl-enzyme complex (also known as an enzyme–protein complex). TGs catalyse diverse reactions that are important for cell viability (Figure 1). After the formation of the acyl-enzyme complex and the release of free ammonia, the transamidation reaction takes place, resulting in the formation of a covalent isopeptide bond by binding the ε-amino group of lysine (Figure 1a). This reaction leads to cross-linking between glutamine and lysine residues within polypeptide chains. When the acyl-enzyme complex reacts with a primary amine, the amine is bound through a γ-glutamyl bond, resulting in the enrichment of the protein chain with hydrophobic or hydrophilic fragments (Figure 1b).

The deamination reaction of the γ-carboxamide group of glutaminyl residues in proteins/polypeptides (forming the acyl-enzyme complex) occurs when water acts as the substrate, and the product is glutamic acid (Figure 1c). This reaction is accompanied by the release of ammonia as a by-product. Additionally, TGs can catalyse other reactions that are relevant to their biological functions and have implications in various cellular processes.

Wang et al. [9] demonstrated that the cross-linking reaction between glutamine and lysine occurs faster than acyl transfer and deamidation in any protein-based system. In each of these reactions, free ammonia is produced as a byproduct. To control the progress of TG-catalysed reactions, measurement of the generated ammonia can be used as an indicator of enzyme activity. Another method of assessing the activity of the enzyme is by quantifying the production of the dipeptide ε-(γ-glutamyl)lysine using HPLC, as described by Rezvankhah et al. [16] and Wang et al. [9]. This dipeptide formation represents the covalent isopeptide bond. The control of protein cross-linking reactions can also be performed by evaluating the molecular weight of the resulting peptide polymers using electrophoresis. Rheological methods, which involve measuring the viscosity or structural parameters of the product, can also be employed to monitor the cross-linking reaction [17].

## 3 TGs of animal, plant, and microbial origin

TGs exist in various forms and can be derived from animal, plant, and microbial sources. These enzymes can be intra- or extracellular, which contributes to their diverse and versatile functions. Enzymatic activity of TG has been detected in the tissues and body fluids of animals, fish, plants, and microorganisms [18,19]. Protein structures involved in various metabolic reactions need to interact with each other. These physical interactions are commonly expressed as hydrogen bonds, ionic bonds, or van der Waals forces. However, these intermolecular bonds and forces are relatively weak. To form stronger covalent connections, plant, animal, and microbial cells produce enzymes called TGs. This enzyme, along with aliphatic polyamines, such as putrescine, spermidine, and spermine, participates in processes such as photosynthesis in plants, embryogenesis in mammals, cell division in microorganisms, and is also involved in the stress response and ageing in each of the mentioned organisms [20,21].

## 4 Animal transglutaminases (TGa)

### 4.1 Types of TGa

Various types of TGs are present in most animal tissues and body fluids, which participate in numerous biological processes such as blood clotting, skin keratinisation, erythrocyte membrane stiffening, and wound healing [22]. In mammalian tissues, up to eight different types of TGs have been identified and characterised, including the blood component Factor XIII [5]. Until the late 1990s, TG for industrial use was primarily obtained from animal tissues [23].

In the early stages of TG research, the focus was on the protein cross-linking involved in human blood clotting. Subsequently, more attention was given to the cross-linking of other proteins. TG from guinea pig liver was the most commonly used enzyme for such studies. Additionally, partially purified TG from bovine blood (Factor XIII) and human placenta were also investigated [24].

In addition to the presence of the discussed enzyme in mammalian blood plasma, the presence of TG has been discovered and purified from erythrocytes. However, the relatively small amount of human blood and its origin make this enzyme less suitable for large-scale applications in food. The abundant availability of bovine and porcine blood makes isolating TG from these sources a more practical solution. By utilising two types of TGs – from animal blood and of microbial origin – cross-linking experiments with seven proteins (α-lactalbumin, β-lactoglobulin, BSA, casein, haemoglobin, myosin, and glycinin) revealed significant differences in substrate recognition and cross-linking rate [25]. These differences among TG types can be attributed to their roles in natural processes. The fact that these enzymes are capable of cross-linking proteins other than their natural substrates indicates they can be utilised in various applications where enzymatic protein cross-linking is desired instead of chemical cross-linking. Applications can be focused on the development of protein polymers with modified functional properties, as well as direct applications in complex systems such as food. Depending on the number and types of proteins involved in the reactions and the specific cross-linking needs, the most appropriate source of TG can be selected [26,27].

In the context of applications related to food products or protein ingredients, the cross-linking of the described substrates appears promising, especially since protein cross-linking can have a significant impact on functional properties. The possibility of using different types of TG to achieve the desired effect can be intriguing, particularly because the rate and number of cross-links formed will vary depending on the type of enzyme used. In issues related to erythrocyte TG, such as self-cross-linking, the need for reducing agents, and challenges in the purification process, limit the potential utilisation of this enzyme [3]. Blood plasma TG offers better possibilities, although purification to a homogeneous enzyme preparation may not be necessary. A good example of the application of partially purified TG derived from plasma is in the meat processing industry, where it is used in combination with fibrinogen to create a system enabling the cross-linking of meat components [28,29].

Due to its limitations in terms of application and production, TG of animal origin has not found widespread use in food processing. The complex procedures for extracting and purifying the enzyme from the tissues or body fluids of slaughtered animals and other livestock (such as cattle, pigs, fish, and poultry) result in a high price for the enzyme (around $80 per 1U), making the use of TG impractical [22,30]. The current price of this enzyme is $189.96 per 1U [31]. Ethical considerations (animal welfare) have also played a role in limiting its use. In addition to the high price, TG has other limitations. Commercially extracted Factor XIII (in Europe) from bovine and porcine blood causes red discoloration of the final treated product, which is an undesirable visual characteristic. Another drawback of Factor XIII is that it is obtained in an inactive form. Thrombin (a serine protease enzyme found in blood plasma) is required to activate the enzyme [22]. The dependence on Ca^2+^ is another limitation of TG of animal origin. For the aforementioned reasons, none of the TGs of animal origin have been positively received by public opinion [20,23]. Currently, TG extracted from the liver of domestic fowl is only used as a laboratory preparation, for example, for comparing TG activity.

Despite the lack of homology in amino acid composition, TG of animal origin exhibits biochemical properties and catalytic activity very similar to that of TG of microbial origin [7]. When considering TGs of animal origin, it should be noted that they participate in many physiological processes, including spermatogenesis, bone formation, and the development of the central and peripheral nervous systems. TG in the human body is responsible for catalysing numerous processes such as blood clotting, epidermal keratinisation, tissue healing, and the development of the salivary glands, heart, and lungs [21,24].

### 4.2 TG in the y-glutamyl cycle of humans

TG plays an important role in the y-glutamyl cycle, which is responsible for the synthesis and breakdown of glutathione (GSH) – the main intracellular antioxidant. TGs in the animal body indirectly participate in cell protection against reactive oxygen species [32]. There are at least eight proteins from the TG family, including TG1, TG2, TG3P, TG4P, TG5, TG6, TG7, and TG8P. In their work, Samelak et al. [21] reported the identification of at least nine genes encoding TGs in the human genome. It is worth noting that TGs encoded by these genes have analogues in other animals, even in invertebrates. This fact can be explained by the high conservativity of these genes, resulting in low genetic variability, as well as similarities in the secondary and tertiary structures of TGs [33]. Among the eight human TGs, only TG1 and TG5 exhibit catalytic activity in the y-glutamyl cycle, and this varies in level. Kinetic analysis has shown that TG1 is 46 times more active than TG5 in the breakdown of GSH. Genetic analysis has also shown that TG1 dominates among the family of TG proteins, and its deficiencies can cause a severe disease called glutathionuria, which involves serious growth disorders. Defects in the gene encoding TG5 do not cause glutathionuria symptoms [34]. Impairments in the activity of this group of enzymes can lead to serious diseases such as Huntington’s disease, Alzheimer’s disease, or Parkinson’s disease [21]. Another TG, TG2, exhibits activity on cell surfaces and in the extracellular matrix. This enzyme participates in wound healing, tissue mineralisation, and skin barrier function. TG2 has the ability to cross-link high-calcium matrix proteins, which has a crucial impact on proper bone formation [35,36].

### 4.3 TG and coeliac disease (CD)

In addition to deficiencies in TG, the products generated by the enzyme-mediated protein reactions can also have immunogenic and potentially pathogenic effects, as seen in individuals suffering from CD. This disease is a complex condition influenced by various factors such as infections, food, medications, vaccinations (against influenza and HPV), toxins (such as aflatoxins), heavy metals, abdominal and gynaecological surgeries, hygiene levels, tobacco smoking, alcohol consumption, socioeconomic status, and lifestyle. These factors also contribute to an imbalance in the gut microbiome, characterised by the abundance of *Proteobacteria* and a reduction in *Lactobacillus* populations, which are associated with the development or increased incidence of CD [36,37,38].

The literature suggests that additives in processed food affect the gut microbiome and increase intestinal permeability, thereby contributing to autoimmune responses. In 2015, a hypothesis was proposed suggesting that TG may play a role in the pathogenesis of CD, and subsequent studies have provided further evidence supporting this hypothesis [38]. Given that gluten is rich in glutamine (∼30%) and contains lysine (<2%), which serve as the donor and acceptor of acyl groups, respectively, it is an ideal substrate for post-translational modification through transamidation or deamidation. The current literature indicates that consumption of products containing mTG leads to reduced immunoreactivity and intolerance to the proteins present in bread products [2,37].

### 4.4 TG and the human gut microbiome

In addition to endogenous TGs and those added to food, there is another possibility for the production in the human body – the gut microbiome. Its dimensions, composition, diversity, cellular products, and activity have led to its characterisation as a “*superorganism*.” One component of the microbial metabolite represented as mobilome is the secretion of TG. By using sequence search programs, thousands of bacteria encoding TGs can be detected, with the majority belonging to the *Firmicutes phylum* [39]. TGs are secreted by microorganisms as a survival factor. Analysis of gut fluid gave positive results for TG activity, although the authors did not differentiate its origin [40]. In this context, mTG plays a crucial role in the survival of gut bacteria. It demonstrates anti-protease capabilities, inhibits the activity of antimicrobial peptides, possesses emulsifying properties, acts against phagocytosis, and influences the equilibrium between Th1 and Th2 immune responses [41,42]. According to the current knowledge, no endogenous TG activity has been reported in the duodenal lumen, in contrast to the intestinal mucosa [43]. Extraintestinal sources of TGs are much broader. Probiotics belonging to prokaryotes serve as a reservoir of microbiological TG, representing an active payload that influences the processes occurring in the intestinal space. It is known that microorganisms can transfer virulence factors to other microorganisms through horizontal gene transfer (HGT) [43,44]. HGT refers to the sideward transfer of genetic material between individual unicellular or multicellular organisms. Unlike vertical gene transfer, which occurs between generations, HGT facilitates the movement of genetic sequences across distant species, typically through mechanisms such as transformation, transduction, conjugal transfer, or specific gene transfer agents. While the evolution of Eukaryotes is primarily guided by vertical inheritance, bacteria and archaea predominantly rely on HGT as a significant means of genome diversification and acquiring new functions. This process is vital for their survival under the influence of natural selection and for achieving successful reproduction [45].

Microbes that inhabit food and coexist without causing harm offer a significant pathway for HGT. For instance, *Lactococcus lactis*, found in the Spanish traditional raw milk product, has been observed transferring genetic material to other lactococci and enterococci [46]. Another distinct facet of industrial food production involves food additives, which have recently been suggested as potential contributors to autoimmunity by influencing the integrity of tight junctions in the intestinal epithelium. There is a suggestion that mTGs could play a role in the progression of CD [47]. Recent findings indicate that these enzymes are immunogenic in individuals with CD, serving as a novel marker that mirrors intestinal damage [48]. In certain pathogens like *Streptococcus suis*, the enzyme functions as a virulence factor, providing anti-phagocytic traits. Whether mTGs in other bacteria might also contribute to virulence remains unclear. Considering the substantial HGT occurrences within the gut ecosystem and their capacity to infiltrate human cells, these enzymes could potentially participate in autoimmune diseases [49].

An additional enzymatic payload of TG delivered to the intestinal lumen comes from pathobionts. Besides their ability to post-translationally modify proteins and disrupt tight junction integrity, they contribute a pathogenic payload represented by mTG. Environmental pathogenic microorganisms can exchange mobile elements with microorganisms residing in the human gastrointestinal tract. Increasingly, contamination of surface waters and soils with animal waste/faeces is being observed, posing a global health threat. It is not surprising that commonly consumed plants and vegetables contain TG. Even if their sequence homology to mTG is not high, they are capable of cross-linking peptides, including gluten. They have even been suggested as possible factors in the pathogenesis of CD, initiating the process in the intestinal lumen. Examples of mTG carriers include apples, soy, bean sprouts, fodder beets, rosemary leaves, Jerusalem artichokes, spinach leaves, and green peas. Routinely consumed plants, fruits, and vegetables may exhibit TG activity. It has been shown that polyols, intensively used in the processed food industry for protein coating, biofilm formation, product gelling, and bio-packaging, improve the thermal stability and half-life of mTG, thereby increasing the enzyme’s ability to form cross-links [50]. As can be observed, both animal and mTGs can coexist in the same organism. mTG will be described in more detail in subsequent chapters.

### 4.5 Plant transglutaminases (TGp)

In the case of plant-derived TGs, it has also been confirmed that multiple TGs can function within a single plant. The role of this enzyme group in plants is equally important as it involves growth and development processes. These enzymes participate in cell division, protein storage, and control of cell membrane stability. TGp can be classified based on the specific substrates associated with their respective locations. In the case of plant cells, molecular weights are used instead of abbreviations to designate the enzymes. For instance, within chloroplasts, the stromal TG acts on RuBisCo as its substrate, and has a molecular weight of 150 kDa. Chloroplast thylakoids have different substrates, mainly the 39 and 58 kDa LHC II proteins. Another organelle rich in TGs is the cytoplasm, where the substrates are actin and tubulin, and the characteristic TG for these substrates has a molecular weight of 80 kDa. High-molecular-weight proteins present in the cell wall serve as substrates for the 58 kDa TG [21]. Thus, TGp plays diverse roles in plants, by being involved in the regulation of cellular and structural processes, cell division, and maintenance of membrane integrity and cellular structures. Both animal and plant-derived enzymes require the presence of calcium ions to fulfil their functions. By binding to the enzyme molecule, Ca^2+^ ions indirectly contribute to controlling the availability of substrates at the binding sites. The indirect influence in this process can be explained by the conformational change of the protein molecule that occurs after the binding of the calcium ion, resulting in the exposure of the amino acid residues in the catalytic centre of TG. This characteristic feature of the enzyme allows the formation of characteristic cross-links only when there is a sufficient concentration of calcium ions in the vicinity of TG. Serafini-Fracassini and Del Duca [51] confirmed in their studies that a calcium ion concentration of above 3 mM is necessary for protein cross-linking reactions by TG. In their research on pea roots and leaves, the scientists observed that such a concentration of Ca^2+^ is normally present in the cytosol, enabling TGs to function properly. It is worth noting that during programmed cell death, the calcium resources increase, which can be utilised by living cells in an environment with a reduced concentration of these cations [52]. The dependence of TGa on calcium ions has been investigated using erythrocytes as an example. Lorand and Graham [36] described a study in which they eliminated Ca^2+^ ions from the reaction environment, leading to irreversible changes in the erythrocyte membrane (shape deformation). Increasing the concentration of the missing cations stimulated TG2 to form cross-links between spectrin and ankyrin in the red blood cell membrane.

An important difference between animal and plant-derived TG is the dependence of the latter on light [52,53]. This statement is supported by the findings of Campos et al. [53] who detected the activity of a 58 kDa TG in maize chloroplasts. High activity was observed in the presence of light, while in darkness, the activity of TG58 was minimal. The literature reports also indicate the inhibitory effect of magnesium ions and thiol groups on TG activity. The disruption of disulfide bridges may also contribute to the improvement of TG activity in animal cells [21,54].

## 5 mTGs

### 5.1 Characteristics of mTGs

**Figure 2:**
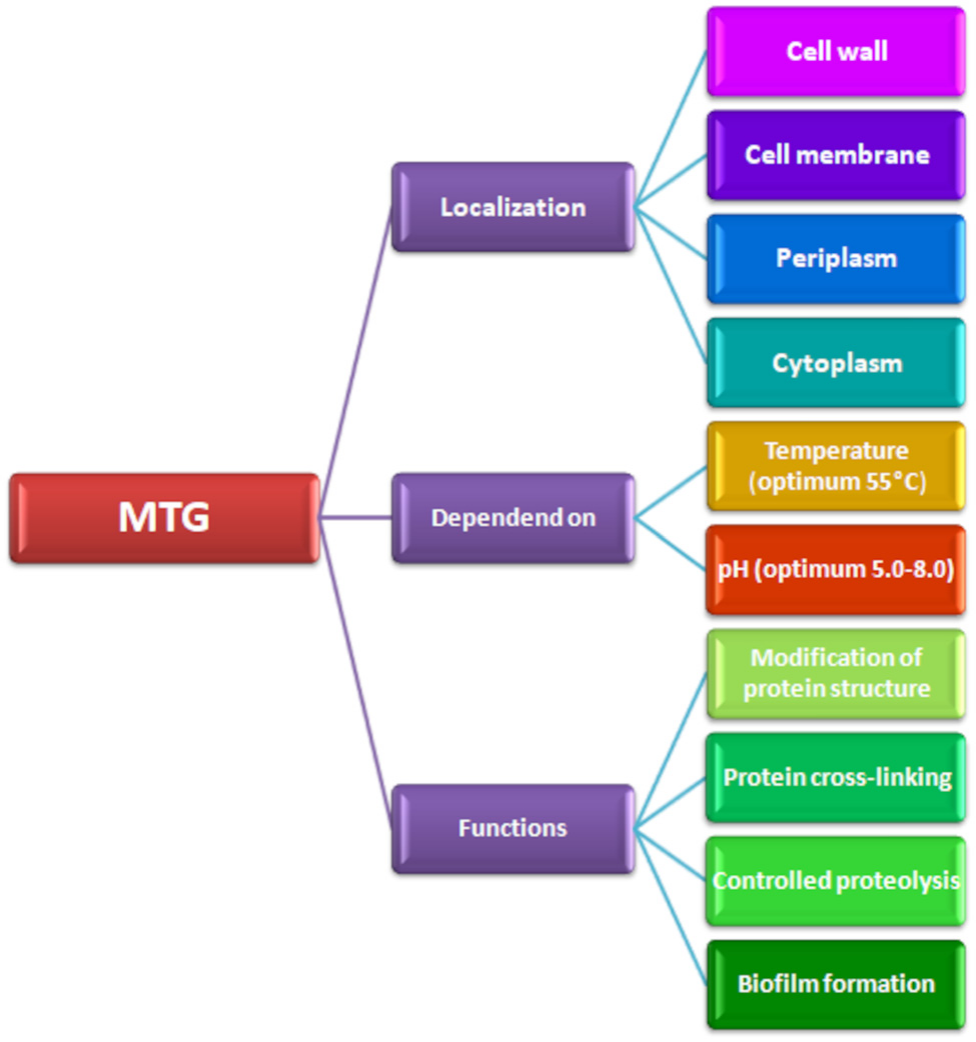
Presentation of possible localisation, functions, and properties of microbiologically derived TGs.

None of the TGp or TGa enzymes have been commercialised or publicly accepted, which has led to the search for a suitable commercial source. TG of microbial origin (Figure 2) was first isolated from the *Streptomyces mobaraensis* strain (previously classified as *Streptoverticillium mobaraense*) [5,6]. Ando et al. [6] examined approximately 500 strains isolated from various soil samples, and among the microorganisms tested, the ability to synthesise this enzyme was demonstrated by the *Streptoverticillium* S-8112 strain. Studies conducted by Ando et al. [6] showed that this enzyme can also be synthesised by other bacteria of the *Streptoverticillium* genus, such as *S. griseocarneum*, *S. cinamoneum* subsp. *cinnanoneum*, and *S. mobaraensis* [30]. The development of research and analytical techniques enables the isolation of additional strains capable of producing TG (Table 1). Moreover, some of the known microorganisms undergo genetic modifications to create strains characterised by efficient synthesis of this enzyme [55].

**Table 1:** mTG activity obtained by various microorganisms

No.	Species	Strain	Substrates and conditions	mTG activity	Reference
1	*Streptoverticillium mobaraensis*	S-SI12	Polypeptide, glucose, K_2_HPO_4_, MgSO_4_; temp. 30°C; 48 h	2.5 U/mL	[6]
2	*Streptoverticillium mobaraensis*	CBS 20778 (WSH-Z2)	Starch, peptone, yeast extract, MgSO_4_, K_2_HPO_4_, KH_2_PO_4_; pH 7.0; temp. 30°C; 7 days	2.90 U/mL	[56]
3	*Streptoverticillium mobaraensis*	CBS 20778 (WSH-Z2)	Starch, glucose, peptone, yeast extract, MgSO_4_, K_2_HPO_4_, KH_2_PO_4_; pH 6.8; temp. 30°C; 48 h	3.30 U/mL	[57]
4	*Streptoverticillium ladakanum*	NRRL-3191	Xylose, peptone, yeast extract, sodium caseinate, MgSO_4_, KH_2_PO_4_, Na_2_HPO_4_; temp. 26°C; 120 h	0.3 U/mL	[58]
5	*Streptomyces mobaraensis*	NRRL B3729	A mixture of soybean meal and wheat bran (1:9), MgSO_4_, KH_2_PO_4_, NH_4_NO_3_, NaCl, CoCl_2_, MnSO_4_, ZnSO_4_, FeSO_4_; pH 6.0; temp. 30°C; 7 days	800 IU/mg	[59]
6	*Streptomyces platensis*	NRRL 2364	Red bean, MgSO_4_, KH_2_PO_4_, NH_4_NO_3_, NaCl, CoCl_2_, MnSO_4_, ZnSO_4_, FeSO_4_; pH 6.0; temp. 30°C; 7 days	5,100 IU/mL	[59]
7	*Bacillus circulans*	BL 32	Soluble starch, peptone, yeast extract, MgSO_4_, K_2_HPO_4_, KH_2_PO_4_; pH 7.0; temp. 30°C; 24 h	0.69 U/mL	[60]
8	*Streptomyces* sp.	CBMAI 1617 (SB6)	2.5% soybean meal, 2% potato starch, 0.1% glucose, 1% bacteriological peptone, 0.4% KH_2_PO_4_·7H_2_O MgSO_4_·7H_2_O; pH 7.0; temp. 30°C; 96 h	6.07 U/mL	[61]
10	*Penicillium chrysogenum*	—	Dextrose 0.5%, tryptone 0.2%, and starch 0.5%, incorporating lysine, glutamine, lysine-glutamine	7.81 mU/mg	[62]

The intensive development of research on mTG began in the late 1980s [54]. Currently, TG is mainly obtained using the species *S. mobaraensis*. Protein sequencing has revealed that the primary structure of mTG consists of 331 amino acids in a single polypeptide chain [22,63]. The secondary structure is composed of eight β-sheets surrounded by 11 α-helices, while the tertiary structure has a disc-like shape with the active site located in the cleft of the apoenzyme, consisting of the cysteine-aspartic acid-histidine triad, which is key to its cross-linking efficiency [64]. mTG possesses the same catalytic triad as TGa but in a different sequence order (mTG: Cys-Asp-His; TGa: Cys-His-Asp) [22,23,64,65].

mTGs have a low molecular weight. mTG is a single polypeptide with a molecular weight of approximately 38 kDa, without any carbohydrate or lipid groups, making it about half the size of TGa [22,66]. The isoelectric point (pI) of mTG has been determined to be pH 9.0. Although the pI may vary due to glutamine auto-deamidation during cultivation, the reported data for pI 8.9 and pI 8.0 indicate that mTG is typically positively charged at pH 6.0. The optimal pH for mTG activity ranges from pH 5.0 to pH 8.0. Ando et al. [6] demonstrated that mTG exhibits activity even at pH 4.0, indicating that the enzyme is stable over a wide pH range. Acidification below pH 4.0 causes enzymes from *Streptomyces* spp. to precipitate. The optimal temperature for mTG enzymatic activity has been found to be 55°C (for 10 min at pH 6.0), and the enzyme loses full activity at extreme temperatures (40 and 70°C) [64]. The addition of carbohydrates such as maltodextrin, sucrose, mannose, trehalose, or reduced GSH increases the thermal stability of TG. At temperatures close to 0°C, TG retains its full enzymatic activity [7]. Recent studies provide information that the optimal temperature for TGs is 37°C, and activity is typically lost at temperatures above 50°C [50]. On the other hand, a study by Yokoyama et al. [22] demonstrated that mTG can also exhibit activity at 10°C, suggesting that mTG is active over a broad temperature range, which is important for its application in various industrial sectors. Such a wide temperature range, as well as pH range, may indicate the need for more detailed studies to provide precise information depending on the specific microbial strain being investigated.

mTG does not require calcium ions for activation, unlike TGs of animal and plant origin. This is highly significant in an industrial context because some proteins (such as casein, myosin, and soy globulin) are sensitive to calcium ions, and their presence can lead to undesired protein precipitation [22]. The activity of mTG increases in the presence of ions such as Co^2+^, Ba^2+^, and K^+^. mTGs are inhibited by ions such as Zn^2+^, Cu^2+^, Hg^2+^, and Pb^2+^, which bind to the thiol group of cysteine in the active centre [67]. mTG also exhibits high sensitivity to disulfide reagents and electrophilic inhibitors, such as cystamine (2,2′-dithiobisethanamine), 5,5′-dithiobis(2-nitrobenzoic acid), *N*-ethylmaleimide, iodoacetic acid, and p-chloromercuribenzoate. It has also been shown that TGs, including MTG, are inhibited by the natural macromolecule melanin in *Streptomyces lavendulae*, most likely through the nucleophilic addition of the catalytic thiolate of cysteine to the inhibitory molecule’s o-quinone groups [50].

### 5.2 Production of mTG by microorganisms

The biological functions of TG in microorganisms are not fully understood. It is known that this enzyme is associated with the cell wall, and it has been suggested that it may be involved in protein cross-linking, the formation of cross-links between cell wall proteins (in *Streptomyces*, *Candida albicans*, *Saccharomyces cerevisiae*), and the cross-linking of proteins in spore coats (in *Bacillus* bacteria) [25]. In *Candida* and *Saccharomyces* yeasts, TG forms cross-links of structural glycoproteins. In *Phytophthora* (oomycetes), it plays a role in the pathogenicity of this organism. In the case of the algae *Chlamydomonas*, mTG combines polyamines with glycoproteins [55]. Scientists suggest that the acquisition of mTG production capability may have been initiated by HGT, as there are no conservative gene clusters, and the TG gene environment differs depending on the species. The absence of mTG genes in the genomes of most *Streptomycetes*, especially in strains such as *Streptomyces coelicolor* A3(2) and *Streptomyces griseus* subsp. *griseus* IFO 13350, may further indicate HGT [50].

A strain of *Streptoverticillium* S-8112, derived from soil, achieved the highest mTG activity of approximately 2.5 U/mL upon reaching the stationary growth phase. Specific antibodies revealed the export of mTG as a zymogen when *S. mobaraensis* DSM 40847 was allowed to grow for 24 h. In shaken flasks, activation began after 35–45 h and typically concluded 2 days later. However, based on numerous experiments under similar conditions, complete processing of the zymogen is a rare event, and cultures lasting over 70–90 h lead to the proteolytic degradation of mTG. Prolonged cultivation further promotes the formation of inactive mTG through autocatalytic deamidation of glutamine, gradually lowering the pI of the TG from pI 8.9 or pI 8.0 to pI 6.4. The recent purification of two mTG variants differing in charge is consistent with this observation [68].

The metalloprotease TAMP cleaves the zymogen mTG on the amidic side of phenylalanine, leaving behind a tetrapeptide, FRAP, at the mature N-terminus. The tetrapeptide is removed by the specific tri/tetrapeptidyl aminopeptidase (TAP), which is secreted by *S. mobaraensis* at the early stage of cultivation. The flexible loop of FRAP and its specific recognition sequence also allow chymotrypsin and trypsin to remove the propeptide by cleaving the peptide bonds Phe-Arg and Arg-Ala. However, TAP only cleaves the remaining tripeptide from RAP-mTG. Processing of AP-mTG fails for unknown reasons, although APpNA is a useful substrate for TAP. A leucine/phenylalanine aminopeptidase, secreted by *S. mobaraensis*, is likely involved in the final processing of mTG [43].

As mentioned earlier, the active site of all TGs includes a catalytic triad consisting of cysteine, histidine, and asparagine, which catalyses two consecutive reactions, similar to cysteine proteases. When the substrate, typically a protein, serving as a glutamine donor, enters the catalytic core of TG, the cysteine thiol attacks the γ-carbonyl group, forming a tetrahedral oxyanion (most likely on the residual side) if the leaving amide group is directed towards histidine or asparagine. An unknown proton relay system then facilitates the elimination of ammonia and ammonium ions to form an intermediate thioester [38].

Investigation of the structure of the binary transition state is not possible as the thioester hydrolyses in the absence of primary amines. Therefore, it remains unclear where the energy for thioester bond formation comes from. The high-energy complex then reacts with amino nucleophiles, which can be the lysine residues of the donor protein, polyamines, or other primary amines of any charge. It is worth noting that cross-linking occurs through a transition state involving three proteins. After the formation of the second oxyanion intermediate, the catalytic cysteine thiol is displaced, and the TG dissociates from the protein conjugate or cross-linked proteins [69].

## 6 Economic benefits

The use of microorganisms in TG production offers increasing economic benefits. The costs associated with microbial synthesis are significantly lower compared to the costs of producing TG from animal sources. However, these are still costs that hinder the widespread use and full commercialisation of this enzyme. One of the major cost factors in obtaining mTG is the culture media, which accounts for approximately 30% of the total costs [7]. To achieve broad commercialisation of mTG, industrial enzyme production requires cheaper feedstocks [70]. Therefore, in recent years, attempts have been made to minimise this cost segment by using waste biomass as microbial substrates, generated by industries such as agriculture. Fatima et al. [31] used wheat bran as an alternative carbon source for *S. mobaraensis* bacteria in solid state fermentation. Another approach aimed at increasing the cost-effectiveness of mTG production is the design and optimisation of cultivation processes. By combining these two approaches, Fatima et al. [31] achieved a four-fold increase in enzyme activity compared to that before process optimisation. Fatima and Khare [12] and Wang et al. [55], using submerged fermentation, obtained enzymes with activities ranging from 2.0 to 5.7 IU/mL. The available literature indicates an increasing number of publications on the biosynthesis of TG. For example, studies conducted by Ryszka et al. [71] demonstrated that the most suitable parameters for obtaining mTG included a medium pH in the range of 6.5–7.0 and nitrogen sources in the form of amino acids, corn steep liquor, and yeast extract. The activity of TG produced by *S. mobaraensis* after 30 h of cultivation reached 2.0 U/mL. The inoculation medium contained 2.5% oat flakes. More examples of the microbial biosynthesis of TG are provided in Table 2.

**Table 2:** Genetically modified microorganisms capable of producing TG

No.	Host strain	Donor strain	Substrates and conditions	mTG activity	Reference
1	*Yarrowia lipolytica* Po1h	*Streptomyces hygroscopicus* WSH03-13	Modified PPB medium (glucose, yeast extract, NH_4_Cl, KH_2_PO_4_, MgSO_4_, thiamine); pH 6.0; temp. 28°C; 200 rpm; 5 days	5.3 U/mL	[85]
2	*Streptomyces lividans* TK24	*Streptomyces hygroscopicus* WSH03-13	R2YE agar or liquid medium (glycerol, peptone, yeast extract, MgSO_4_, K_2_HPO_4_, KH_2_PO_4_, CaCl_2_); temp. 30°C; 200 rpm; 2–3 days	5.73 U/mL	[80]
3	*Corynebacterium glutamicum* YDK010	*Streptomyces mobaraensis* IFO13819	MMTG medium (glucose, MgSO_4_, NH_4_SO_4_, KH_2_PO_4_, FeSO_4_·7H_2_O, MnSO_4_·4H_2_O, thiamine hydrochloride, biotin, DL-methionine, CaCO_3_; pH 7.5; temp 30°C	627 mg/L	[86]
4	*Pichia pastoris* X33	*Streptomyces mobaraensis*	BMGY medium, citric acid medium (glycerol, NH_4_HPO_4_, MgSO_4_·7H_2_O, KCl, CaCl_2_·2H_2_O), biotin pH 5.0; temp. 28°C	9,120 U/L	[83]
5	*Streptomyces mobaraensis* TX1 mutant Sm2-1	*Streptomyces mobaraensis* TX1	Glycerol, yeast extract, peptone, MgSO_4_·7H_2_O, K_2_HPO_4_·3H_2_O, (NH_4_)_2_SO_4_; pH 7.4	37.5 U/mL	[87]
6	*Escherichia coli* BL21 (DE3)pLysS	*Bacillus amyloliquefaciens* DSM7	TB broth (tryptone, yeast extract, glycerol, KH_2_PO_4_, K_2_HPO_4_)	37 mU/mg	[25]
7	*Pichia pastoris* GS115	*Escherichia coli* DH5α	Yeast extract, peptone, K_2_HPO_4_, yeast nitrogen base without amino acids, biotin, CH_3_OH 28°C; 250 rpm; 96 h	4.40 mg/L; 0.88 U/mg	[88]
8	*Bacillus subtilis* 168	*Streptomyces mobaraensis* CGMCC 4.5591	Corn starch, peptone, urea, K_2_HPO_4_·3H_2_O, KH_2_PO_4_, MgSO_4_, NaCl, saccharose	29.6 U/mg	[82]
9	*Komagataella phaffii* X-33	*Streptomyces netropsis* EF195356.1	1% yeast extract, 2% peptone, 1.34% YNB, biotin, 1% glycerol,	26.17 U/mg	[89]
10	*Lactococcus lactis* NZ9000	*Bacillus amyloliquefaciens* BH072	GM17 medium supplemented with chloramphenicol at a final concentration of 5 μg/mL	28.6 U/mg	[43]
11	*E. coli* Transetta (DE3)	*E. coli* DH5α	Soluble starch, peptone, yeast extract, MgSO_4_·7H_2_O, K_2_HPO_4_·3H_2_O_2_, KH_2_PO_4_	4.99 U/mL	[90]

The use of TGs in industry offers several other advantages. First, it reduces the energy demand during the production process. Furthermore, the waste generated from TG utilisation is non-toxic and biodegradable, making it environmentally friendly [72]. It is worth noting that one drawback of using enzymes in industry is that certain production conditions may be unsuitable for optimal enzyme performance. Important aspects for optimal enzyme efficiency include temperature, pressure, pH, and the presence of potential inhibitors [73]. Microbial sources are preferred in industrial enzyme production. mTGs are readily available and easy to produce. Another advantage is that the growth rate of microorganisms is much faster compared to animals or plants. Most importantly, in addition to optimising the production process of natural mTGs, genetic and metabolic engineering can be employed to increase the efficiency of enzyme synthesis in microorganisms. Genetic or metabolic engineering can also aid in obtaining enzymes that can function under diverse production conditions (pH and temperature) [74].

## 7 Genetic modification of microorganisms and their significance in mTG production

Genetic modification of microorganisms plays a significant role in the production of mTG. Currently, the industrial use of TG is primarily derived from the bacterium *S. mobaraensis*. This mTG is the only one with Generally Recognised as Safe (GRAS) status [75]. However, the quantities of TG obtained using this single strain are not sufficient for global-scale mTG utilisation. In order to achieve more efficient and cost-effective enzyme production, and thus expand the commercialisation of mTG, genetic manipulation of microorganisms has been pursued [76]. So far, expression systems have been developed for bacteria, such as *Escherichia coli* [77,78], *Streptomyces lividans* [79,80], *Corynebacterium glutamicum* [81], and *Bacillus subtilis* [82], and for yeast species such as *Yarrowia lipolytica* [80] and *Pichia pastoris* [19,83] (Table 2). Increasing the quantity of enzyme produced involves both mutagenesis of the original production strains and genetic manipulations among strains.

Until recently, *E. coli* was considered unsuitable for inducing TG expression. The expression levels in *E. coli* were low, and the microorganism did not secrete the enzyme into the culture medium – it was produced intracellularly (in the form of intracellular inclusion bodies). In such cases, to obtain purified and soluble enzyme, the inclusion body cells need to be disrupted, and the insoluble mTG needs to be refolded, making the entire process time-consuming and uneconomical. Liu et al. [84] developed a method for the direct production of soluble and active mTG by *E. coli*. After purification, the activity of the recombinant enzyme was 22 U/mg [84]. Another successful expression of the mTG gene in *E. coli* resulted in a specific activity of approximately 37 mU/mg protein. Duarte et al. [25] used genetic engineering techniques to clone the gene encoding mTG from *Bacillus amyloliquefaciens*. Mu et al. [82] obtained an enzyme with an activity of 29.6 U/mg by cloning the gene encoding mTG from *S. mobaraensis* into *B. subtilis*.

Genetic manipulations, in addition to increasing production efficiency, also aim to improve the activity and thermostability of TG [8]. Thermostability, solubility, and, in some cases, enzyme efficiency, can be manipulated by altering the amino acid sequence. Improving the thermostability of the enzyme expands its applications and positively impacts the durability and stability of the resulting products [8,91]. Increased thermostability was achieved after a single round of mutagenesis leading to the substitution of one amino acid in the enzyme [91]. Yokoyama et al. [92] obtained mTG with a specific activity of 45 U/mg, which was 1.7 times higher compared to the wild-type enzyme (22.7 U/mg). The researchers used an innovative method called wash-rotational mutagenesis [8]. Yin et al. [76] applied mutagenesis and targeted genetic modification to the bacterium *S. mobaraensis*. The *S. mobaraensis* DSM40587 strain was treated with atmospheric and room-temperature plasma (ARTP) in 25 repetitions, producing approximately 3,000 mutants in each repetition. Among the mutant strains obtained, the mutant smY2019 was selected, which showed a 5.5-fold increase in mTG activity (19.7 U/mL). Further steps involved integrating two additional expression cassettes containing the PsmY2019 promoter with the genome of the smY2019 strain. The resulting strain, smY2019-3C, showed a 103% increase in mTG production (40 U/mL) compared to the smY2019 strain. ARTP utilises a plasma stream containing various activated chemical species for DNA mutagenesis. This new mutation system can induce a higher level of microbial mutation compared to traditional methods [76].

## 8 Concerns and controversaries of mTG usage in the food industries

According to the available literature, the daily consumption of mTG through processed food products can vary, reaching levels of up to 15 mg. Each kilogram of processed product containing mTG typically contains around 50–100 mg of the enzyme. Intriguingly, a noteworthy connection can be observed between the rising yearly consumption of commercial enzymes added to processed bakery items and the concurrent increase in the incidence of CD over the past four decades. While this relationship suggests an association, a direct cause-and-effect relationship has not been definitively established.

A growing body of information is being collected regarding mTG’s classification as an allergen in occupational settings [41,93]. In their analysis, the Aaron and Torsten [41] compiled a variety of research that substantiates TG’s status as an allergen. They noted that products containing wheat/gluten treated with MTG exhibit immunoreactivity, raising doubts about its safety. The supporting argument is safety assessments, producers have primarily conducted *in vitro* tests on cell lines and animal models such as mice, guinea pigs, and rats. However, these evaluations have not been extended to encompass normal or gluten-dependent human conditions. Ultimately, the responsibility for demonstrating mTG’s safety lies with its manufacturers and suppliers [45,75].

Formally, mTG is classified as a processing aid, allowing it to avoid being categorised as a food additive. mTG does not require labelling, as indicated by the manufacturer’s assertions. According to them, the enzyme’s activity is neutralised or its substrates are depleted during the production process of the end product, resulting in the absence of TG in the final item. This aligns with its role as a processing aid in accordance with regulation 89/107/EEC, making labelling unnecessary [41]. Numerous authors express concerns and caution against the inclusion of mTG in industrially processed foods, raising doubts about its safety and its potential role in the pathogenesis of CD. In Europe, some regulatory authorities have taken steps to caution the public about the safety of mTG in food and have recommended labelling of the enzyme. For example, in Switzerland, food treated with TG requires authorisation, and the labelling must provide information about the reconstitution process and the nature of the used “glue.” In Germany, the BFR Bund authorities have indicated that there is a potential clinically relevant risk for CD patients from mTG. Appropriate labelling of foods produced using mTG would enable these patients to avoid uncertainties that the scientific community has yet to clarify [41].

Drawing from the disruption of intestinal tight junctions caused by gluten, the posttranslational modifications of gluten peptides induced by mTG, and the heightened immunogenicity of the resulting mTG-gliadin neocomplex (mTG-gliadin complexes can represent an offending autoantigens), a hypothesis has been proposed. This hypothesis suggests that mTG could potentially serve as a novel environmental element in the initiation and progression of CD. However, its role in non-coeliac gluten-sensitive conditions remains uncertain, posing a significant challenge for future investigations [47,48].

## 9 Food safety and TG

Due to the increased use of TG in food production, health concerns have emerged, emphasising the need for regulations informing people about the safety of consuming products containing this enzyme. Almost a quarter of a century ago, at the turn of the twentieth and twenty-first centuries, scientists from Japan (Motoki and Seguro) demonstrated that the only difference between proteins subjected to the influence of TG and native proteins is the number of connections between the lysine and glutamine residues (G–L) [23]. This chemical modification also occurs during the heating of protein-rich food, such as cooking, generating G–L bonds. In this regard, humans have been consuming food rich in G–L residues since the discovery of fire and cooking. Although not scientifically proven, the safety of modified G–L bonds can be assumed through the long-term consumption of G–L molecules in thermally processed food. As for the absorption and bioavailability of such proteins, 99% of isopeptides in the gastrointestinal tract are metabolised into glutamate. At the same time, an American research team led by Bernard examined the mutagenesis and risk of microbiological toxicity of TG associated with its addition in food preparations tested on experimental animals. The study concluded that mTG does not pose a health risk, as no mortality, morbidity, or signs of toxicity were observed at doses of 2 g/kg body weight [23]. It is important to note that mTG is safe for human consumption and has been recognised by the Food and Drug Administration as a GRAS substance since 1998 [94].

Following discussions in food articles about the use of TG, an important aspect is the safety for those people with allergies. Poulson and Bindslev-Jensen [66] performed sequence matching in protein databases to compare the amino acid sequence of mTG derived from the *S. mobaraense* strain with all known allergens at that time. As a result of their research, the scientists did not find any homology with any allergen type that met the decision tree requirements (matching six adjacent amino acids). It is worth noting that a match of five adjacent amino acids was discovered between mTG and the major allergen Gad c1 from cod (known for its thermal stability). Further studies were conducted regarding the potential allergenic activity of mTG. Targeted screening of mTG was performed using sera from patients with cod allergy, as well as direct inhibition of RAST. No binding between patients’ IgE and mTG was observed, leading researchers to conclude that there are no concerns regarding potential allergic reactions in humans related to fish products.

There is some evidence of enhanced nutritional properties in enzymatically modified food using TG. According to studies, adding mTG to soybean extract for tofu preparation led to protein modifications that increased satiety and reduced the allergenicity of soy proteins. In another application related to allergenicity, shrimp products processed using TG showed reduced allergenicity due to protein glycosylation catalysed by this enzyme [64].

## 10 Utilisation of TG on an industrial scale

The industrial production of TG for large-scale applications can be challenging and costly, especially when using animal-derived TG. Industrial production of TG relies on bacterial sources (including actinomycetes) as they are easier and faster to cultivate, and the yield of the TG produced is significantly higher. mTG exhibits the lowest substrate specificity and provides the greatest possibilities for protein component cross-linking [95].

The first producer of mTG on an industrial scale was the Japanese company Ajinomoto Co (still active in the market). The first food product manufactured using mTG was a frozen fish concentrate called “Surimi” in Japan [23]. The preparation of Surimi fish paste consists of two stages. The first stage, also known as “binding,” occurs at temperatures below 40°C and leads to the formation of a semi-transparent and elastic gel. The second stage, carried out at high temperatures (>80°C), is necessary to strengthen the gels. Gelation is caused by the cross-linking of myosin induced by endogenous Ca^2+^-dependent TGase present in the walleye pollock, the fish used to prepare Surimi. It has been demonstrated that the addition of exogenous TGs, both blood isoforms and microbial isoforms, during the binding process is beneficial for obtaining Surimi paste with increased elasticity and tensile strength. Furthermore, all fish products restructured using mTG exhibit high whiteness, which is considered an important characteristic for consumers. Therefore, the application of TG is desirable for the efficient restructuring of Surimi-based products [2].

mTG has a potentially wide range of applications in food products, especially those structured based on proteins. The enzyme can enhance various properties of food proteins without affecting the pH, colour, taste, or nutritional value of the food [9]. It acts as a texturising agent, imparting firmness to finished food products, improving thermal stability, and the water-holding capacity of food (meat, fish, seafood, dairy, bakery, and pasta products) [66].

The modification of food proteins by TG helps protect the lysine in food proteins from adverse chemical reactions, thereby increasing the shelf life of food. Furthermore, by cross-linking other proteins containing essential amino acids, proteins with higher nutritional value can be obtained. TGs can be used for encapsulating lipids or lipid-soluble substances, creating heat and water-resistant films. Scientific data also suggest that TGs improve the quality of wool in felting, bleaching, and shrink resistance processes. Incorporating different functional groups into the glutamine residues of proteins using TG is highly useful as it enhances and increases the final utilisation of the protein.

## 11 Commercial TG preparations

Commercial TG preparations on the market vary due to the different industrial applications of this enzyme. All preparations contain the enzyme obtained from bacteria of the species *S. mobaraensis* [96]. The preparations differ in their accompanying additives (maltodextrin, gelatin, sodium caseinate, and polyphosphate salts), which characterise their suitability for use in specific product groups. Some preparations are used for the production of sausages or hot dogs, while others are used for fermented dairy products. Among the very few companies involved in the production of commercial TG enzyme preparations, notable ones include Ajinomoto Co. Inc. (Japan) – ACTIVA^®^ enzyme preparations [97], BDF Ingredients (Spain) – BDF PROBIND^®^ preparations, PMT TRADING Sp. z o.o. (Poland) – SAPROVIA and POLSEVIA preparations [7,97].

## 12 TG in the baking industry

In the process of creating yeast-leavened products using low-quality wheat flour, the dough frequently lacks the desired stability. This type of dough fails to retain the carbon dioxide gas produced during fermentation. As a result, it is quite common to introduce oxidizing agents to the flour in order to enhance its ability to withstand stretching. Due to the desire to minimise the use of synthetic chemicals in food production and instead utilise natural helpers such as enzymes, the challenge emerged in enhancing the dough’s resilience without resorting to inorganic compounds. Researchers discovered that TG enhances the dough’s resistance, especially in the case of yeast-based dough made from wheat flour, in a manner akin to the effects of potassium bromate [38]. In the baking industry, this enzyme is used to improve the quality of flour, bread texture, volume, and the texture of cooked pasta [98]. From a nutritional standpoint, rice flour contains many valuable nutrients, including protein, fibre, and vitamins E and B. Studies conducted by Gujral and Rosell [99] have shown that the addition of TG to rice flour improves the rheological properties of the dough by increasing the content of triglycerides.

The applications of TG in cereal proteins, particularly in wheat proteins (globulins, glutenins, gliadins, and prolamins), have attracted significant interest from the baking industry. The cross-linking of wheat proteins through the action of TG greatly influences the characteristics of products, determining their quality, functional properties, and rheological behaviour, including stability, elasticity, resilience, water absorption (with the appropriate pore size and dough volume). The cross-linking reaction promotes aggregation and polymerisation, leading to the formation of polypeptide networks with diverse viscoelastic properties [100].

mTG positively affects the structure of low-gluten wheat bread, as well as its yield and volume. In the study by Diowksz and Leszczyńska [101], it was shown that microbial enzymes contribute to the reduction in immunoreactivity. These findings have led to preliminary investigations of the use of low-gluten flours in the production of gluten-free products. The enzyme is also proposed for use in blocking allergenic, protease-resistant peptides in soy proteins [102]. The enzyme can exert beneficial effects during bread production, comparable to those of traditional chemical oxidising improvers. When using small amounts of TG, a positive impact on the crumb and crust quality of bread, as well as on the rheological and physicochemical properties of the dough, has been observed [103]. The release of certain gluten peptides resulting from mTG activity may also influence the modulation of the bread microbiota during storage, thereby increasing the durability of the final product [104].

TG is used in the baking industry to catalyse gluten cross-linking in wheat, thereby improving the quality, structure, volume, stability, texture, and durability of the dough. TGs are currently used in baking technologies to create connections between polypeptide chains of prolamins. Previous studies have shown that transglutaminase has a positive impact on dough stability and volume, as well as on improving the baking quality of weak flour, consequently affecting bread texture [105]. Sadowska and Diowksz [24] suggest that adding TG to a gluten-free mixture containing buckwheat flour increases the moisture content of the bread crumb and reduces overall baking losses. TG improves the rheological properties of the dough and ensures the proper pore size and elasticity of the bread after baking. Additionally, TGs have been shown to enhance water absorption by the dough. Modification of wheat flour proteins by TG increases dough elasticity and resilience, as well as bread volume by 14% compared to traditionally prepared dough [106].

An interesting and unconventional application of TG is the attempt to restore the functionality of the gluten network in wheat flour obtained from grains damaged by insects. In many countries, a significant portion of crops that have been damaged does not meet standards and is unsuitable for further processing or production, resulting in waste. Research exploring the possibility of recovering lost crops is highly promising for farmers facing losses and for food producers. Damaged wheat flour treated with TG exhibits parameters similar to undamaged flour [107].

Currently, the microbial isoform of the enzyme is widely used in the production of pasta in Japan. Pasta treated with TG has higher firmness compared to untreated pasta, and this increases with the amount of added enzyme. It has been shown that reactions catalysed by mTG also effectively influence the functional properties of baked goods through protein aggregation and polymerisation, leading to the formation of a polypeptide network with viscoelastic properties required for bread baking. Gaspar and De Góes-Favoni [108] reported several studies on the physical properties of bakery products treated with TG, as well as the potential of this enzyme in modifying rice proteins. There are numerous reports on the use of TGase for modifying gluten proteins. Wheat globulins, glutenins, and gliadins were more effectively modified by transglutaminase than prolamins from oats, corn, and rice. The gelation properties of wheat gluten were significantly improved by thermal treatment followed by TG-catalysed cross-linking reaction [2].

Isolated soy protein (ISP) is widely used as an important ingredient in Asian diets and processed foods due to its nutritional value and functional properties. ISP consists of glycinin (11S) and β-conglycinin (7S), which make up about 70% of the total protein content. These globulins are good substrates for TG activity. The influence of TGase on the properties and microstructure of ISP films formed using different plasticisers (glycerol, sorbitol, and a 1:1 mixture of glycerol and sorbitol) has been investigated. Cross-linking with this enzyme proved to be an effective method for improving the casting properties of films with all the tested plasticisers [64].

Tofu, a typical soy product, is prepared by coagulating soy proteins with the addition of Ca^2+^ and Mg^2+^ and/or glucono-delta-lactone. The first step in tofu production is the extraction of soy milk from soybeans and its coagulation. Coagulation or gelation of soy is the most important stage in tofu production. Traditionally, nigari, a sediment from seawater rich in minerals such as magnesium and calcium chlorides, has been used as a coagulant. Modern producers use TG (together with nigari) to obtain tofu with a smoother and firmer texture (silken tofu). The addition of TGase before nigari makes the coagulation process milder and easier to control. The same effect is achieved by adding glucono-lactone, but its use imparts an undesirable acidic taste to the final product [64]. Tofu is a popular food consumed extensively in Asian countries; however, its shelf life is typically very short due to its soft and smooth texture, which hinders sterilisation. Introducing mTG into its processing provides an advantage in texture control and improves its quality, resulting in a product with better consistency, silky texture, and the ability to tolerate temperature fluctuations [3]. Studies by Xing et al. [109] demonstrated that the addition of TG during tofu production results in protein modification that increases satiety and reduces the immune response to soy proteins [64,110].

Rice protein enzymatic cross-linking has received limited scientific research attention. The availability of commercial concentrates and isolates from rice bran is limited due to the complex nature of rice bran proteins. On one hand, the crude protein content in rice (5–10%) is significantly lower compared to other grains. On the other hand, it is important to note that the lysine content, which is one of the substrates suitable for protein cross-linking reactions with TGase, is over 50% higher compared to wheat. Rice proteins alone are not capable of forming a strong enough structure to retain gas produced during fermentation. The addition of hydrocolloids (hydroxypropylmethylcellulose; HPMC) to the dough recipe allowed for the production of bread from rice flour. Protein cross-linking using TGase by Gujral and Rosell [99] enabled the partial replacement of HPMC and improved the viscoelastic properties of the dough. According to Gujral and Rosell [99], the expansion of rice flour applications has significant potential, mainly due to the global popularity of this raw material. Rice proteins cross-linked with TGase to form a stable network and improve nutritional properties offer promising prospects for the production of new products intended for individuals with gluten intolerance, CD, or other specific dietary requirements [102].

## 13 TG in the meat industry

TG has gained interest in the food industry for its ability to enhance the texture of minced meat, resulting in a firmer steak-like structure. Restructuring meat products provides greater firmness and minimal quality loss during cooking. The cross-linking of proteins and other components in the gel system brings about changes in the protein fraction of food matrices, leading to improved texture, thermal denaturation stability, emulsifying properties, gelation, and increased water-binding capacity. The application of TGase yields a final product with organoleptic properties similar to conventional meat in terms of aroma, texture, appearance, and taste [64].

In addition to its positive impact on product texture, TG enables strong cohesiveness of meat blocks without the need for heat treatment or the addition of salt and phosphates. Reducing sodium content in meat products is an important health concern, and to meet these requirements, the meat industry focuses on developing techniques to reduce the use of salt in processed meat products without compromising quality. Strategies such as the use of TG can be employed in low-sodium meat products. The impact of TG, fibrin/thrombin (fibrimex), alginate, and their combinations on the quality of low-salt cooked meat has been studied. The results indicate that the combination of fibrin/mTG improved the texture properties of low-salt ground beef [3,111].

The use of TG in meat processing significantly improves the texture of the final product, resulting in increased firmness, among other benefits. Additionally, this enzyme enhances the texture of homogenised sausages made from pork, beef, or poultry. The addition of TG enables the utilisation of lower-quality raw materials, such as collagen, blood proteins, and mechanically deboned meat, to produce meat products with higher nutritional value by complementing them with amino acids (e.g., exogenous lysine) [2]. Several studies have reported the application of TG in meat products [64,111]. The enzyme can be used over a wide range of temperatures, from 10 to 50°C. Some of these studies also demonstrated that supplementing the enzyme can increase gel strength in meat products and have a positive impact on the development of proteins in pork, beef, chicken, and fish. As meat products are highly proteinaceous, myofibrillar proteins have a significant influence on their textural quality. Actin and myosin, which constitute the majority of myofibrillar proteins, are important substrates for TG and can be polymerised through its addition, improving the textural properties of structured meat products. The use of TG has created new technological possibilities for the production of finely and coarsely comminuted sausages and processed meats. Instead of using high-quality meat, lower quality raw materials and additives, such as defatted milk powder, soy flour, or wheat flour, can now be employed. The interaction of the enzyme with the proteins in these raw materials produces products that do not differ in appearance, consistency, aroma, taste, or nutritional value from analogous products made exclusively from high-quality meat [2,112].

In the meat industry, the use of TG has expanded beyond the reconstruction of lower quality minced or mechanically deboned meat into steaks or larger meat cuts. Research in the industrial application of TG focuses on current dietary trends and consumer preferences for healthy food choices. An example of such an application is the production of meat analogues. The production of meat analogues is a response to the growing demand for alternative food products among vegans, vegetarians, and health-conscious consumers who seek substitutes for animal products [5]. Producing meat analogues poses a challenge for food manufacturers due to the difficulties in replicating the texture and taste of the base products. Studies focus on combining plant proteins (e.g., pea protein) with TG to improve their structural properties, potentially replicating the structure of animal-based products. In the study by Moreno et al. [113], the impact of TG on the structural quality of pea protein isolates was examined. The structure of the analogue was improved, and a reduction in the levels of biogenic amines (histamine and tyramine) was achieved [5]. A modern approach to producing meat analogues involves the use of 3D printers. The study by Wen et al. [114] aimed to develop a beef analogue that mimics the textural properties of the raw material using 3D printing and the addition of TG. In addition to developing the recipe itself, the study tested various methods of heat treatment (steaming, microwaving, baking, and frying) for the created analogue. As a result, technological guidelines for the production of such meat analogues were developed [114].

## 14 TG in the dairy industry

In the highly competitive market of dairy products, companies constantly strive to capture consumers’ attention. Improving the quality and functionality of products in this industry is an ongoing race, where promoting biofunctional properties holds an increasingly prominent position. One of the most promising strategies for promoting biofunctional properties in dairy products is the cross-linking of milk proteins using TG. The application of TG can be an effective strategy for enhancing the nutritional and technological properties of dairy products while lowering production costs by reducing the amount of fat and stabiliser in the final product. This enzyme has the ability to create intra- and intermolecular covalent cross-links between two amino acid residues in the structure of milk proteins. Both casein and whey proteins, such as α-lactalbumin and β-lactoglobulin, are excellent acyl donors and/or acceptors for TG, although there are some differences between them regarding cross-linking reactions [64].

In the cascade reaction, TG catalyses the cross-linking of κ-casein (κ-CN) and β-casein (β-CN) before proceeding to the cross-linking of serum albumin, α-lactalbumin, αs1-casein, αs2-casein, and β-lactoglobulin (β-LG). In this particular case, caseins seem to be easily cross-linked due to their flexible, randomly coiled structures and the absence of any disulfide bonds. Whey proteins, due to their compact globular structure, tend to cross-link less efficiently. β-LG is more susceptible and exhibits a higher cross-linking rate than α-LA, with β-LG capable of cross-linking with the reduction of its disulfide bonds, while α-LA can be cross-linked without reduction [2].

The polymerisation of milk proteins is an essential process in the production of certain dairy products. TG is added to milk proteins to catalyse the polymerisation process, aiming to enhance the nutritional and functional properties of dairy products [115]. Lorenzen et al. [116] reported the use of TG in the dairy industry to prevent syneresis and impart proper softness and firmness to dairy products. TG-modified casein enables the production of dairy products with improved structure and consistency. The enzyme can be added before or during the fermentation process. Using this method, TG is added to milk for incubation, resulting in yogurts with a homogeneous, firm, and creamy consistency, as well as a smooth and dry curd-like surface, which is a result of reduced syneresis. Such yogurts serve as a base for the production of creams, frozen desserts, ice creams, dairy beverages, and dressings [117]. Furthermore, recent studies have shown that enzymatic cross-linking of milk proteins by TG improves the consistency and microstructure of goat milk yogurt gel [3]. Interestingly, microbiological analyses have demonstrated that enzymatic modification of goat milk proteins plays a positive role in the survival of probiotic bacteria used for yogurt production.

An intriguing application of TG is its use to enhance the survival of lactic acid bacteria during spray drying and to increase microencapsulation efficiency [5]. The traditional method of spray drying lactic acid bacteria is fast and inexpensive, but unfortunately, the survival rate of bacteria is only around 31%. Treating a mixture of soy proteins and milk proteins with TG increased the survival rate of *Lactobacillus bulgaricus* to 84.7% (during spray drying) [118].

Another type of dairy product with enormous potential for the application of TG is cheese. Cheese holds a significant position in the dairy industry’s commercial market. Global cheese production reaches approximately 19 million tons annually, accounting for 35% of total milk consumption [119]. One of the major challenges faced by the cheese sector is production efficiency, which ranges from 9 to 20% depending on the type of cheese, and for fresh farmer’s cheese, it falls between 10 and 13%. Cheese yield is a crucial parameter from an economic standpoint for producers [120]. In the study conducted by Fox et al. [119], cheese yield is defined as the kilograms of cheese produced from 100 kg of milk with specific levels of fat and protein. For example, the typical yield for cheddar cheese (with a moisture content of 39% and a fat content in dry matter of 50%) is 10 kg of cheese per 100 kg of milk standardised to protein and fat levels of 3.3 and 3.6%, respectively [119].

In addition to the economic benefits, increasing cheese production efficiency helps reduce environmental pollution by generating smaller amounts of by-products (whey). Enzymatic modification of milk proteins is a crucial element in strategies aimed at enhancing cheese production efficiency. TG and phospholipases are primarily utilised for this purpose, as their application does not lead to sensory defects but rather optimises the efficiency and desired properties of the product. The cross-linking of milk proteins using mTG is gaining increasing popularity. It improves the rheological and physical properties of acid milk gels, as well as production efficiency by reinforcing emulsifying capacity, water-binding abilities, and solubility [120,121].

Three models for the production of natural cheese with TG are proposed [94]:

(a) Addition of TG to milk, heating the milk for pasteurisation, and deactivation of the enzyme, followed by the addition of rennet.
(b) Addition of rennet to milk, followed by the addition of TG.
(c) Addition of TG to milk simultaneously with rennet.

Cozzolino et al. [122] examined these models in their study. The results indicated that transglutaminase plays a significant role in improving cheese yield and properties. It was observed that adding TG before rennet prevented milk coagulation, while simultaneous addition of the enzyme and rennet significantly reduced cheese hardness. The potential advantage of TG treatment is considered to be the improvement in texture and water-holding capacity of both fresh and ripened cheese. It has been demonstrated that protein cross-linking enhances curd yield, and the addition of mTG during the manufacturing process enables the production of protein-enriched cheese with increased firmness [95].

## 15 TG in the pharmaceutical Industry

Increasing consumer awareness of health and nutrition, which are undoubtedly interconnected, has led to growing demand for bioactive compounds, particularly antioxidants [123]. One such compound is curcumin, a low molecular weight polyphenol that exhibits strong antioxidant properties. Curcumin has positive effects on the treatment of certain chronic diseases by reducing oxidative stress, making it of interest to scientists and pharmaceutical companies involved in nutraceuticals. There are also reports of promising possibilities for the use of curcumin as a bioactive agent in food packaging systems [124]. Unfortunately, the poor water solubility and lack of stability of curcumin pose challenges in its direct application. In their study, Xia et al. [125] examined the use of curcumin with improved bioavailability in Pickering emulsions based on oleogels. A Pickering emulsion is one stabilised by solid particles accumulated at the droplet surfaces. This type of emulsion has gained significant interest among researchers in recent years due to its high potential for applications in fields such as medicine, food industry, and petroleum industry [126]. To create a curcumin-containing Pickering emulsion, the researchers chose lactoferrin as the emulsifier. Lactoferrin is a single-chain glycoprotein with antimicrobial properties and the ability to scavenge free radicals and regulate cell growth. TG was necessary in the experiment, as it facilitated protein cross-linking reactions, enabling the formation of lactoferrin particles (LF-TG). The resulting LF-TG particles acted as stabilisers for the formed emulsion. The optimal parameters for obtaining TG -induced lactoferrin particles through cross-linking were a pH of 8.0, TG concentration of 100 U/g LF, a temperature of 50°C, and a cross-linking time of 2 h [125]. This study confirms the potential application of TG in creating Pickering emulsions, which, in turn, can enhance the efficiency of delivering nutraceuticals.

Preparations containing TG can offer solutions to numerous technological challenges associated with food efficiency and texture. It is widely known that the use of phosphates in meat processing technology on a large scale has been abandoned due to health concerns. The available literature emphasises the importance of reducing phosphates in meat processing. In response, various enzymatic preparations of microbial origin containing TG derived from the *S. mobaraensis* strain are available on the market. TG enables the neutralisation of texture changes caused by freezing in raw materials such as chicken meat or chicken fillings. Other ingredients containing TG are also used in the production of salami, as they enable faster product ripening. The use of TG in the food industry, regardless of its source, represents a natural technological method. Enzymatic modification of food ingredients has a greater chance of acceptance by the food industry compared to commonly used chemical methods.

## 16 Conclusion

Although 30 years have passed since the discovery of mTG and the current trend of genetic engineering (developing highly efficient gene expression systems using strains such as *E. coli*, *Corynebacterium* sp., *Pichia* sp., and even *Bacillus* sp.), there is still a need for isolating new microorganisms, optimising growth media, and fermentation procedures to obtain TG with higher activity. Biotechnological advancements enable metabolic engineering of microorganisms to produce tailored TGases with unique properties. However, a complete understanding of the impact of TGs with different properties on the functionality of food systems is still lacking. Currently, commercial TG is produced through fermentation by *S. mobaraensis*, although there are increasing reports of new TGs from other species of actinomycetes. Unfortunately, this system has certain limitations, including low efficiency and problems associated with protease presence (which can hydrolyse the target proteins). Therefore, it is necessary to develop an efficient biosynthesis system for mTG without these limitations. The cross-linking reaction catalysed by the enzyme described can improve the physical properties of various protein-based food products and contribute to the delivery of high-quality, health-promoting new food. From the perspective of advanced protein resource utilisation, the contribution of TG to the food industry will continue to grow. Moreover, this enzyme can be useful in fields beyond the food industry. It is expected that TG will continue to drive innovation in multiple industries, including increased production using new technologies, protein engineering modifications, and the search for TGs from novel sources.

Despite advertisements and publications asserting its complete safety, the enzyme is recognised as a known occupational allergen, immunogenic substance, and potentially pathogenic factor in CD. If substantiated, it could emerge as a novel environmental trigger for CD. Until further information is available from ongoing research, it is recommended that any applications of mTG in commercial food processing or baking be disclosed on packaging labels to ensure transparency for consumers. Gluten-free products are already acknowledged for potential contamination. The addition of mTG could further elevate risks for gluten-sensitive populations.
